# Co-creation to scale up provision of simplified high-quality comprehensive abortion care in East Central and Southern Africa

**DOI:** 10.1080/16549716.2018.1490106

**Published:** 2018-07-04

**Authors:** M. Klingberg-Allvin, S. Atuhairwe, A. Cleeve, J. K. Byamugisha, E. C. Larsson, M. Makenzius, M. Oguttu, K. Gemzell-Danielsson

**Affiliations:** a Department of Women’s and Children’s Health, Karolinska Institutet, Stockholm, Sweden; b School of Education, Health and Social Studies, Dalarna University, Falun, Sweden; c Makerere University College of Health Sciences, Kampala, Uganda; d Department of Public Health Sciences, Karolinska Institutet, Stockholm, Sweden; e Kenya Medical and Education Trust (KMET), Kisumu, Kenya

**Keywords:** Unsafe abortions, maternal mortality, Eastern, Central, Northern Africa

## Abstract

Universal access to comprehensive abortion care (CAC) is a reproductive right and is essential to reduce preventable maternal mortality and morbidity. In East Africa, abortion rates are consistently high, and the vast majority of all abortions are unsafe, significantly contributing to unnecessary mortality and morbidity. The current debate article reflects and summarises key action points required to continue to speed the implementation of and expand access to CAC in the East, Central, and Southern African (ECSA) health community. To ensure universal access to quality CAC, a regional platform could facilitate the sharing of best practices and successful examples from the region, which would help to visualise opportunities. Such a platform could also identify innovative ways to secure women’s access to quality care within legally restrictive environments and would provide information and capacity building through the sharing of recent scientific evidence, guidelines, and training programmes aimed at increasing women’s access to CAC at the lowest effective level in the healthcare system. This type of infrastructure for exchanging information and developing co-creation could be crucial to advancing the Sustainable Development Goals 2030 agenda.

## Background

The World Health Organization (WHO) estimates that 44 percent (25.1 million) of the approximately 56 million abortions that occurred annually between 2010 and 2014 were unsafe [1]. The proportion of unsafe abortions was higher in countries with restrictive abortion laws, such as in the East, Central, and Southern Africa (ECSA) health community [1]. Restrictive abortion laws, poverty, gender inequality, and stigmatisation contribute to inequitable and inadequate healthcare access, causing higher proportions of unintended pregnancy and unsafe abortions in ECSA [1,2]. Inadequate access to comprehensive abortion care (CAC) impedes women’s sexual and reproductive health and rights (SRHR). Previous research shows that some healthcare providers report hesitancy in providing such care and have judgmental attitudes towards women-seeking abortions [3,4]. This hesitancy may be explained by a conceptual conflict between human rights and societal norms, which also causes misconceptions [4]. Such misconceptions create fear of being stigmatised and discourage women from seeking adequate care [5]. The WHO’s technical guidelines on safe abortion from 2012 emphasise the simplification of CAC [6]. An example of simplified care is telemedicine, which should be considered as an alternative service delivery channel for medical abortion [7]; another example is the task shift to nurses and midwives that has occurred in post-abortion care [8,9], which was a pragmatic response to the shortage and uneven distribution of physicians in low- to middle-income countries. Because midwives are key in CAC, scaling up their involvement is an important strategy in expanding access to care. Implementing task sharing within CAC should be a priority but will only be possible if training and continuous support are provided.

Expanding access to CAC and ensuring women’s SRHR are imperative if the ECSA countries wish to contribute to Sustainable Development Goals (SDGs) three (good health and wellbeing) and five (gender equality) that are directly linked to providing high-quality sexual and reproductive health for women and adolescent girls. To address the issue of unsafe abortion and limited CAC access in the region, a number of workshops were held in Kampala, Uganda, in March 2016 and October 2017. The 2016 workshop participants identified both barriers to and facilitators of implementing CAC in East Africa. Abortion stigma was identified as a major barrier to the implementation of quality CAC; participating healthcare providers were also acknowledged as being unaware of established clinical abortion care recommendations [10].

The focus of the 2017 workshop was to facilitate improvements in the quality of CAC and to create discussions about co-creation, exchange, and capacity building in ECSA to improve access to CAC. The long-term goal of the workshop was to create a network consisting of researchers and abortion care providers in Africa, similar to that of the International Federation of Professional Abortion and Contraceptives Associates (FIAPAC). Such a platform would engage professionals working in the field of abortion and contraception in Africa by providing support, capacity building, and knowledge sharing and creating networking and collaboration opportunities. The workshop further created an opportunity to disseminate recent findings in the region and to discuss CAC implementation strategies, including post-abortion care and contraception.

Workshop participants included collaborating partners in Sweden and ECSA with specific scientific knowledge and expertise in the field of CAC, representatives from various ministries of health, the International Federation of Gynecology and Obstetrics (FIGO) working group on the prevention of unsafe abortions, several non-governmental organisation (NGO) partners in development, and the media. NGOs involved in CAC in the region were invited to share their experiences and to help form a capacity-building platform. All participants received policy briefs and abstracts in advance of the workshop to facilitate constructive discussions. During the interactive sessions, participants worked together to develop an action plan for how to utilise the evidence and to implement guidelines in the participants’ particular settings. Participants also identified potential barriers to and facilitators of the implementation process.

The outcome of the workshop was the identification of sustainable solutions to improve CAC in the region: (1) focus on local capacity in building in-service training and education, (2) ensure that curricula are evidence based and updated, and (3) include value clarification and the use of simple, evidence-based procedures for CAC. Strong leadership within healthcare ministries, healthcare systems, and academia is necessary to ensure that policies, standard guidelines, and care are evidence based and stigma free. The engagement of NGOs and civil society will encourage women’s autonomy, safeguard women’s right to evidence-based treatment, and provide respectful, high-quality care. To assure universal access to quality CAC, a regional platform could facilitate the implementation of evidence-based CAC. Using the platform to share best practices and successful examples from the region would help to visualise opportunities and could also identify innovative ways to secure women’s access to quality care within legally restrictive environments. The platform would provide information and capacity building through the sharing of recent scientific evidence, guidelines, and training programmes aimed at increasing women’s access to CAC at the lowest effective level in the healthcare system. This type of infrastructure for exchanging information and developing co-creation could be crucial to advancing the SDG 2030 agenda.

More specifically, the platform would align with the earlier established network – now inactive – titled the ‘FIGO Initiative for the Prevention of Unsafe Abortion and Its Consequences’ and would be scaled up to cover the ECSA region (Ethiopia, Kenya, Mozambique, Tanzania, South Africa, Uganda, and Zambia). The FIGO initiative involves the national societies of obstetrics and gynaecology in less-developed countries in which rates of unsafe abortion or induced abortion are high [11]. The long-term goal of the platform would be to reduce maternal mortality related to unsafely induced abortions and limited access to contraception by implementing CAC focussed on medical abortion and by scaling up access to post-abortion contraception in the region. The platform will disseminate recent scientific evidence and guidelines related to CAC, including post-abortion care and provision in the ECSA region to relevant policy-makers and stakeholders. The platform, which will operate virtually and will enable the sharing of ideas and new evidence and implementation strategies regarding CAC, will be led by senior researchers from ECSA. Yearly workshops will be held in the region, where international researchers as well as the WHO and other relevant actors will be invited to participate.

## Paper context

This debate article highlights important action points regarding abortion care access and policy in East, Central, and Southern Africa (ECSA), particularly Kenya and Uganda. A regional platform could facilitate the implementation of evidence-based comprehensive abortion care (CAC), scale up the exchange of information, and develop co-creation in order to advance the Sustainable Development Goals 2030 agenda in the region.
